# Refining a DEI Assessment Tool for Use in Optimizing Professional STEM Societies for Gender Equity

**DOI:** 10.3389/fsoc.2022.755372

**Published:** 2022-06-14

**Authors:** Gretalyn M. Leibnitz, Jan W. Peters, Rebecca Campbell-Montalvo, Heather Metcalf, Andrea Lucy Putwen, Donald L. Gillian-Daniel, Ershela L. Sims, Verónica A. Segarra

**Affiliations:** ^1^Amplifying the Alliance to Catalyze Change for Equity in STEM Success (ACCESS+), Washington, DC, United States; ^2^ProActualize Consulting, LLC., Moscow, ID, United States; ^3^Katalytik, Christchurch, United Kingdom; ^4^Department of Curriculum and Instruction, Neag School of Education, University of Connecticut, Mansfield, CT, United States; ^5^Women in Engineering ProActive Network (WEPAN), Washington, DC, United States; ^6^Wisconsin Center for Education Research, School of Education, University of Wisconsin-Madison, Madison, WI, United States; ^7^Center for Natural Sciences, Biological Sciences and Chemistry, Goucher College, Baltimore, MD, United States

**Keywords:** science technology engineering mathematics (STEM), equity, professional societies and associations, assessment, survey design, diversity, equity, and inclusion (DEI) tool, professional society self-assessment tool, Equity Environmental Scanning Tool (EEST)

## Abstract

Historic science, technology, engineering and mathematics (STEM) disciplinary cultures were founded in a system that was predominately male, white, heterosexual, and able-bodied (i.e., “majority”). Some societal norms have changed, and so has demand for inclusive STEM engagement. However, legacy mental models, or deeply held beliefs and assumptions, linger and are embedded in the STEM system and disciplinary cultures. STEM reform is needed to maximize talent and create inclusive professions, but cannot be achieved without recognizing and addressing norms and practices that disproportionately serve majority vs. minoritized groups. As leading voices in disciplinary work and application, disciplinary and professional societies (Societies) are instrumental in shaping and sustaining STEM norms. We, leaders of the Amplifying the Alliance to Catalyze Change for Equity in STEM Success (ACCESS+) project, recognize the need to provide Society diversity, equity, and inclusion (DEI) change leaders with tools necessary to foster systemic change. In this Perspectives article, we present the Equity Environmental Scanning Tool (EEST) as an aid to help Society DEI change leaders elucidate legacy mental models, discern areas of strength, identify foci for advancement, and benchmark organizational change efforts. We share our rationale and work done to identify, and, ultimately, adapt a Society DEI self-assessment tool from the United Kingdom. We share background information on the UK tool, content and structural changes made to create the EEST, and an overview of the resulting EEST. Ultimately, we seek to increase awareness of a Society-specific DEI self-assessment tool designed to help Society DEI change leaders advance inclusive reform.

## Introduction

Success in science, technology, engineering and mathematics (STEM) is commonly believed to be the result of objectively determined talent, training, and hard work. In discussing scientific meritocracy, Taylor (2022) explained, “If we have the correct training and skillset for the role combined with enough ambition, if we work hard and do well at our job, then we will be appropriately rewarded via promotion and other recognitions of our career” (p. 729). However, this belief conflicts with the experiences of marginalized individuals, for whom structural biases, discrimination, and inequities exist which blocks them from achieving levels of funding, recognition, and/or reward that are conferred upon peers from majority backgrounds without such barriers, discrimination, and inequities (e.g., McGee, 2020; Bird and Rhoton, 2021). National concern and acknowledgment of barriers faced by women, especially those with additional intersecting identities, and other marginalized groups is longstanding and well-documented (e.g., Valian, 1998; Faulkner, 2007). Still, STEM culture reform to maximize engagement of STEM talent is needed.

STEM disciplinary and professional societies (Societies) are uniquely positioned as agents for DEI reform (National Academy of Sciences, 2005). They play an important role in shaping and maintaining disciplinary culture, fostering STEM awareness and education, and informing standards (Borman et al., 2010; Chanderbhan-Forde et al., 2012). Society members and supporters are drawn from diverse STEM influencers, including academia, industry, and national laboratories. Because Societies shape disciplinary culture and serve diverse stakeholders, they provide multiple levers for STEM diversity, equity, and inclusion (DEI) reform; however, it has not been until the last decade that studies have explored Society members' experiences, and strategies to guide Society DEI change.

One study by Cech et al. (2018) provided evidence of differential experiences of Society majority and minoritized members. Over 16,000 STEM professionals across 14 Societies were studied. Results documented the cumulative disadvantages faced by women, people of Latino, Asian, and African American origin, LGBTQ+ members, and people with disabilities. In comparison to white participants, marginalized respondents reported working harder, being harassed verbally or in writing, and having their work devalued and disrespected. Recent work (e.g., Burnett et al., 2022), pushes for inclusive data collection to enable Societies to better serve underrepresented group members.

Societies aware of experiences of marginalized members can address barriers and promote DEI. Smith et al. (2021) and Campbell-Montalvo et al. (2022a) demonstrated that by providing critical support (e.g., mentoring, networking), Societies increased engineering degree persistence for women and underrepresented engineering undergraduates. Further, the Campbell-Montalvo et al. (2022c) study on LGBTQIA+ undergraduates found that Societies attuned to participants' identities positively impacted retention in STEM. These findings are consistent with the National Academies of Sciences Engineering Medicine (2018) conclusions that many Societies desire to understand the experiences of underrepresented professionals and incorporate strategies to address inequities, but lack critical information, “We can't solve the problems in a vacuum…. We want to identify what kinds of challenges our members are facing and what [we] can do to be a partner and an educator in helping to create actionable steps toward solutions” (p. 5).

In 2017, the Alliance to Catalyze Change for Equity in STEM Success (ACCESS) brought together Minority Affairs Committee (MAC) leaders from five biological Societies to create collective impact around Societies' DEI efforts. These leaders documented and disseminated information on challenges faced as they worked to create systematic inclusive change (Segarra et al., 2020a,b; Primus et al., 2022). ACCESS work demonstrates the value of bringing together Society DEI change leaders to support systematic change.

Building on the work of ACCESS, the recently funded National Science Foundation ADVANCE funded Amplifying ACCESS (ACCESS+) partnership project proposed strategies and tools to help cohorts of STEM Society DEI experts, and “first generation equity practitioners” (Bensimon and Gray, 2020), affect desired DEI change. While ACCESS+ uses multiple tools and strategies [e.g., The Inclusive Professional Framework for Professional Societies (Leibnitz et al., 2022), a monthly Community of Practice, and an annual convening], this paper specifically explores the development of the *Equity Environmental Scanning Tool* (EEST) to help Society DEI change leaders gain a clearer picture of the ways in which the Society may differentially serve majority and minoritized members.

## The EEST: Foundation, Adaptation, and Overview

When exploring Societies' diversity programs and goals, Solebello et al. (2016) found that Society leaders experience tension between the espoused inclusion values, and the drive to protect the exclusivity of the profession and the Societies' history and culture. Addressing this tension requires self-reflection on “deeply held beliefs and assumptions, and taken-for-granted ways of operating that influence how we think, what we do, and how we talk,” (Kania et al., 2018, p. 4), called mental models. Society mental models are manifest in the operations, or functions of the society. They can be reflected, for example, in ideas about what research topics deserve funding, who is qualified to hold Society leadership positions, or whose research or scholarship deserves to be highlighted in convenings or society publications. Because Society mental models represent “how things are done” they can obfuscate the need for, and potentially create counter-pressure to, change.

Self-assessment is a means of uncovering mental models, and is central to ACCESS+'s approach to helping Societies take informed systematic action to advance DEI. Consistent with others (e.g., Ritchie and Dale, 2000), we argue that internally conducted Society self-assessment stimulates critical conversations; affords opportunity to record Society-identified DEI performance benchmarks; provides opportunity for collective recognition of strengths and weaknesses; centralizes data to inform reports and communications; and encourages ownership, broad engagement, and accountability for actionable changes. Society culture reform begins with self-assessment.

After reviewing a number of DEI self-assessment tools, we selected the *Diversity and Inclusion Progression Framework for Professional Bodies* (Framework), created by the Royal Academy of Engineering and Science Council (2021) based in the United Kingdom. The Framework provided UK Societies with means for intra/inter Society DEI conversations; collective benchmarks; rationale for focused action; and for recognizing strengths and identifying blind spots—efforts that have arguably made DEI progress more systematic and robust. We selected the Framework because it was developed by STEM Societies, had a history of meaningful use and application within and between Societies, and had been refined over time (i.e., from an 8-frame model in 2017, with the latest iteration into a 10-frame model in 2021). The Framework not only provided an existing tool specific to the ACCESS+ target STEM Society audience, it provided evidentiary support for the beneficial use and application of such a tool.

### Adaptation

In assessing the Framework's applicability for use with US. Societies, we determined that two main types of changes were needed: first, content changes related to differences in the Society functions between the United Kingdom and the United States; and second, structural changes permitting the assessment to be used as both a discussion tool and a means of measurement. Content changes related to differences in: (a) how professional licenses work and are awarded; (b) distinctions in how study programs in universities are accredited; (c) the important role of US. regional and student chapters; and (d) the role of US. Society disciplinary advocacy in providing comment and contribution on issues of national importance. We also took the opportunity to incorporate recent learning on DEI and academic publishing (Day et al., 2020; Institute of Physics, 2021), and explore the influential role in STEM culture reform Societies have through their choices of partnerships, vendors, and sponsors.

Structural changes dealt with item wording and assessment changes that included the addition of a Likert scale. Additionally, based on preliminary work with the original ACCESS societies, we found that asking Societies to report actual demographic data of their membership created a barrier to completing the tool; consequently, we revised the requirement to reduce demand, yet still provide important demographic information, by clarifying compositional categories (e.g., race/ethnicity, gender) that informed data collection by Societies. Overall, throughout the adaptation and revision process, we consulted subject matter experts and Society DEI leaders to ensure changes.

The EEST is currently undergoing ongoing piloting and refinement with a first ACCESS+ cohort of the original 5 ACCESS biological societies, and a second cohort of 14 predominately engineering societies. Tool refinement efforts are informed by semi-structured interviews, informal interviews, and focus groups with Society cohort change leaders, as well as discussion and outcomes from Community of Practice meetings.

### Overview

The EEST tool is embedded in a process designed to provide integrated support for Society DEI leaders as they guide DEI change. Consistent with prior work on organizational transformation (e.g., Bilimoria and Liang, 2012), formal authorization and support of the Society chief executive officer (CEO) is required as a necessary part of the ACCESS+ cohort application submitted by Society DEI change leaders. Society DEI change leaders, or “Boundary Spanners,” as identified in prior work (e.g., Leibnitz et al., 2021), then receive and orchestrate completion of an electronic version of the EEST over a span of 8 weeks. Upon receiving the completed Society EEST, ACCESS+ provides Society reports and recommendations. DEI Action Plans informed by EEST results are created at an annual ACCESS+ convening (Campbell-Montalvo et al., 2022b). Ongoing support for identified DEI actions (e.g., helping Societies develop their data monitoring and reporting strategies), as well as deeper exploration of EEST results and other cohort driven topics, is provided *via* monthly Society DEI change leaders' Community of Practice meetings.

Specifically, the EEST consists of three parts. Part 1 identifies twelve Functions representing typical operations that Societies may undertake. Societies complete only the Functions relevant to their operations. Part 2 explores Society data collection approaches on diversity representation; and Part 3 offers the opportunity to document DEI progress, challenges, and priorities. Together these three Parts provide functional measurements, identify data collection that needs to be addressed, and promote distillation of organizational narratives so that DEI progress becomes part of the Society's benchmark records. In doing so, the EEST provides a framework for Societies to undertake a rigorous review and reflection of what it is doing, and what it could do, to benefit members (and potential members), stakeholders, the profession, and the discipline.

#### Part 1

Part 1 of the EEST comprises twelve common functions typical of Society operations (see Table 1: EEST Function Titles and Descriptions). Together, the functions collectively represent the core structures of a Society that are central to its performance. These include the people, staff and members in the Society (i.e., Governance & Leadership, Membership), how the Society engages with its members (i.e., Meetings, Conferences & Events, Chapters & Affiliates, Marketing & Communication, Community Outreach & Engagement), how it socializes and recognizes members within the field (i.e., Professional Development, Awards & Recognition, Publishing, Public Policy & Advocacy), and how it operationalizes staff and business relationships (i.e., Employment and Partners, Sponsors, & Vendors). Please note, the numbering of the Functions is not intended to convey DEI priority or importance (e.g., #11 Publications is no less a priority for DEI focus than #4 Professional Development). Additional explanation of the functions can be found in Peters et al. (2021).

**Table 1:** The EEST Part 1 twelve society functions and descriptions.

**EEST part**	**Society function**	**Description**
1.1	Governance & Leadership	Explores how DEI is integrated into the ethos of the Society's leadership, how the Society is governed, and how major decisions are made about its goals and activities.
1.2	Membership	Examines the design and delivery of the Society's membership activities, as well as the experience of its members.
1.3	Meetings, Conferences & Events	Identifies who participates, how they participate, and what they experience during Society meetings, conferences, and events.
1.4	Professional Development	Focuses on professional development opportunities, including skills in leadership and management, networking, and technical certifications/licensure.
1.5	Chapters & Affiliates	Examines the support, development, and activities available for members in chapters, including those active in secondary, postsecondary, and other educational and non-academic settings.
1.6	Awards & Recognition	Identifies the established application and selection policies and procedures by which people apply to, or are nominated for, awards and recognition.
1.7	Marketing & Communication	Considers how the Society communicates with its members and stakeholders and the content that is communicated/marketed.
1.8	Community Outreach & Engagement	Explores how the Society promotes and engages the wider community, public, and other stakeholders in the Society's sphere of influence.
1.9	Employment	Examines how employees are recruited, managed, and promoted in the Society.
1.10	Public Policy & Advocacy	Focuses on how the Society promotes and protects the interests of the discipline and its members.
1.11	Publishing	Explores how the Society manages its publishing processes and produces official publications and journals.
1.12	Partners, Sponsors & Vendors	Considers how the Society selects and works with partners, sponsors, and vendors.

Within each of these 12 Society functions are three sections: (1) Management and Administration, (2) Policies, Procedures and Practices, and (3) Insights and Evaluation. Sections contain between 5 and 15 statements for consideration. We use these three sections to help understand how Societies are embedding DEI into strategies, actions, and impacts. Statements within each section are evaluated on a 5-point Likert Scale (0 = Never; 1 = Rarely; 2 = Sometimes; 3 = Often; and 4 = Always) to assess if, and how often, the case of DEI change is enacted in the operations of the society. A “Not Applicable” option is available for statements that do not apply to both individual statements and whole Functions. An overview of sections and example statements are provided below.

The first section on *Management and Administration* incorporates statements about the composition of leadership groups, DEI professional development in which leaders are engaged, and governance strategies employed by leaders as related to DEI. Previous research shows that *leadership* is key to cultural change within an organization (Bilimoria et al., 2008; Bilimoria and Liang, 2012, 2014; Martins, 2020). Specifically, organizational change is facilitated by leaders' attitudes and approaches, assistance in developing new ways of thinking, and responsiveness to stakeholders (Eckel et al., 2001). An example statement in Section 1 for the “Governance and Leadership” Function is, “The society has a strategic plan that specifically addresses DEI.” Another example is, “The society's process for selection and/or election of Organizational Leadership is transparent, equitable, and inclusive.”

The second section on *Policies, Procedures and Practices* incorporates statements that explore the day-to-day operations and enforcements of policies in which norms and expectations are constructed, and programming is aligned (or not) with espoused leadership goals as related to DEI. An example statement in this section, for the “Awards and Recognition” Function, is “The Awards Team has developed and regularly reviews equitable criteria for selecting awardees.” Another example statement is, “The Awards Team engages with other STEM Professional Societies to develop society DEI good practices.”

The third section, *Insights and Evaluation*, asks respondents to consider statements “tracking key indicators of representation and equity; evaluating programmatic interventions and strengthening the institutional research infrastructure to improve data collection, analysis and use” (Bilimoria et al., 2008) as related to DEI. An example statement in this section, for the “Publishing” Function, is “The Publishing Team has articulated what data it will collect from and about authors and how they will be used to inform inclusive publishing practices.” Another example statement is, “The Publishing Team agrees on what constitutes clear evidence of sustained behavioral and cultural change with regard to its DEI work.”

Once completed, Part 1 statements are averaged for each of the 12 Functions, and three Sections, to help ascertain Society DEI activity. Averages for Functions and Sections are computed and interpreted based on the following five-point scale, going from no activity to transformative:

- *No Activity (Scores* = *0–0.99)*: a case for DEI change has not been made
- *Idling (Scores* = *1–1.99)*: a case for change is emerging, data and insights are starting to be gathered, actions tend to be informal, isolated bottom-up or one-off
- *Developing (Scores* = *2–2.99)*: the case for DEI change is clear, some data are being gathered, responsibility and accountability being formalized, guidelines being developed, activity being launched, connections being made
- *Engaging (Scores* = *3–3.99)*: the case for DEI change is well-established, data are being gathered and shared, sustained senior level support is in place, skills and capabilities being built, activity catching on, high levels of collaboration, clear signs of change
- *Transforming (Scores* = *4)*: the case of DEI change is focused on transforming the culture and systems of the organization. Complex qualitative and quantitative data are being routinely, intentionally, and systematically gathered and shared, high levels of dialogue, collaboration and learning, clear evidence of change in individual behavior and organizational culture

#### Part 2

In Part 2, Society respondents are asked to provide information on how DEI efforts are measured by the Society for each of the 12 Functions covered in Part 1. Actual data are not requested, instead Societies are asked to indicate demographic data collected based on “compositional” categories (e.g., gender, race, ethnicity, disability, sexual orientation, sociometric background).

#### Part 3

Part 3 asks the Society team completing the EEST to answer open-ended questions designed to gather information about areas of Society DEI successes and challenges; intersectional strategies employed in Society Functions, and DEI priorities for the future. Specifically, the open-ended questions include: (1) “In what area of DEI has the society made the most progress?” (2) “Of what organizational DEI efforts are you most proud?” (3) “How are you employing an intersectional approach?” (4) “What are the society's main challenges in making progress on DEI?” (5) “What are the Society's DEI priorities for the next 12–24 months?” and (6) “Is there anything else you feel is important to document about the society to benchmark for future consideration?”

## Conclusion

In our experience, many Society leaders recognize that STEM is better able to address global concerns when diverse talent is welcomed, supported, and retained in the field (Borman et al., 2010). They also recognize that disciplinary excellence is being undermined by (1) loss of talent, (2) lack of equitable advancement, and (3) compromised STEM research, products and services due to lack of inclusivity in approach, design and/or application. STEM Professional Societies are uniquely positioned to address these limitations and act in support of DEI, but can encounter pockets of resistance, and lack resources and/or tools to elucidate and address the existing mental models that protect the history and culture of the Society, resulting in restricting the change desired. The ACCESS+ leaders have identified, adapted and are refining a needed Society DEI self-assessment tool to support Society DEI change leaders. The EEST, paired with the *Inclusive Professional Framework for Societies* (Leibnitz et al., 2022), and supported by a Society cohort-based Community of Practice provide valuable scaffolding for Society transformation. Evaluation results from preliminary work with the first ACCESS+ cohort of five biological Societies indicate that DEI change leaders value the EEST for structured conversations, as well as for raising awareness of the breadth, and depth, of what can be done. Cohort one also valued the professional support from the ACCESS+ team during office hours, and at the Annual Convening (Campbell-Montalvo et al., 2022b). To date, pilot efforts support the EEST's potential efficacy, along with ACCESS+'s programming, to provide systemic support for Society leaders to create consequential DEI change.

## Data Availability Statement

The original contributions presented in the study are included in the article/supplementary material, further inquiries can be directed to the corresponding author/s.

## Author Contributions

JWP and RC-M completed an initial draft of the manuscript. GML and JWP completed the final revision of the manuscript. All authors contributed to review, edit, revision, and/or addition of new material.

## Funding

This material is based upon work supported by the National Science Foundation under Grant No. 2017953.

## Conflict of Interest

GML is the Director of ProActualize Consulting, LLC, a consulting business specializing in applying evidence-based strategies to promote inclusive organizational and disciplinary excellence. JWP is the Director of Katalytik, a consulting business based in the United Kingdom focused on promotion and creation of evidence-based equity and inclusion across STEM education and employment. The remaining authors declare that the work was conducted in the absence of any commercial or financial relationships that could be construed as a potential conflict of interest.

## Publisher's Note

All claims expressed in this article are solely those of the authors and do not necessarily represent those of their affiliated organizations, or those of the publisher, the editors and the reviewers. Any product that may be evaluated in this article, or claim that may be made by its manufacturer, is not guaranteed or endorsed by the publisher.
